# Stereotactic breast biopsies: Radiological-pathological concordance in a South African referral unit

**DOI:** 10.4102/sajr.v26i1.2463

**Published:** 2022-08-26

**Authors:** Natasha Alexander, Ilana Viljoen, Susan Lucas

**Affiliations:** 1 Department of Radiology, Faculty of Radiation Sciences, University of the Witwatersrand, Johannesburg, South Africa

**Keywords:** stereotactic breast biopsy, BI-RADS, breast cancer, digital breast tomosynthesis, radiological-pathological concordance

## Abstract

**Background:**

Stereotactic breast biopsies have become the gold standard for tissue diagnosis in non-palpable, sonographically occult breast abnormalities seen on mammogram. Only limited data exist in South Africa on the correlation between imaging findings and stereotactic biopsy histology.

**Objectives:**

To describe the mammographic findings and histological diagnosis in patients who underwent stereotactic breast biopsy at a referral hospital. In addition, to evaluate the proportion of malignancy in each Breast Imaging Reporting and Data System (BI-RADS) category.

**Method:**

A retrospective review of stereotactic breast biopsies was performed. Imaging characteristics (including BI-RADS category) and histological diagnosis were recorded. Using histopathology, cases were classified as benign, high-risk or malignant.

**Results:**

A total of 131 biopsies, from 123 patients, were included in the study. Most biopsies were performed on asymptomatic patients (79.3%, 104/131). The majority were categorised as BI-RADS 4 and demonstrated calcifications. Histology revealed a malignant diagnosis in 40 (30.5%) patients, a high-risk lesion in 8 (6.1%) patients and a benign diagnosis in 83 (63.4%) patients. There was a stepwise increase in the proportion of malignancy from BI-RADS category 3 to 5. When compared with surgical histology, the stereotactic biopsies demonstrated an overall ductal carcinoma in situ (DCIS) underestimation rate of 10.3%.

**Conclusion:**

Despite resource restrictions, stereotactic breast biopsies performed in a South African context produce radiological-pathological concordance in keeping with BI-RADS guidelines, as well as with local and international studies.

## Introduction

Breast cancer screening has increased dramatically over the past 50 years.^1^ Despite this, breast cancer remains the leading cause of death amongst South African women, and women worldwide.^2,3^ Because of the implementation of screening programmes, radiologists are faced with a wide range of imaging findings (often in asymptomatic patients) and are expected to correctly identify high-risk lesions.^4^ The Breast Imaging Reporting and Data System (BI-RADS) was developed to universalise the mammographic report and guide management. Using all breast imaging modalities available at the time of assessment, the breast lesion or abnormality is given a score from 0 to 6. Category 0 is considered an incomplete investigation. Category 1 is a negative investigation. Category 2 includes only typically benign findings. Category 3 is considered probably benign (≤ 2% chance of malignancy) and imaging follow-up is recommended. Category 4 includes suspicious lesions and is further subcategorised based on probabilities of malignancy (A = low, > 2% but ≤ 10%; B = moderate, > 10% but ≤ 50%, and C = high, > 50% but < 95%). Category 4 lesions are usually referred for tissue diagnosis and form the bulk of minimally invasive breast biopsies. Category 5 lesions are considered highly suggestive of malignancy (≥ 95% probability) and pre-operative tissue diagnosis is usually recommended.^5^

Lesions seen on ultrasound are typically sampled with ultrasound-guided core needle biopsy (CNB).^6^ Stereotactic methods of sampling are widely accepted as the gold standard for mammographically detected, sonographically occult breast lesions.^7,8,9^ This includes two-dimensional (2D) stereotactic biopsy, as well as the more recently developed digital breast tomosynthesis (DBT)-guided biopsy that has proven superior to mammography in detecting architectural distortions.^10^ Digital breast tomosynthesis is now the preferred method for sampling non-calcified, mammographically detected abnormalities that do not have a sonographic correlate.^11,12^ Options for sampling devices generally consist of CNB and vacuum-assisted biopsy (VAB). The latter has proven to decrease procedure time and increase tissue yield, therefore increasing histological accuracy.^13^

To the best of our knowledge, there is only one previous study specifically addressing radiological-pathological concordance of stereotactic biopsies in South Africa: Cupido et al. retrospectively reviewed 67 stereotactic CNBs performed at Addington Hospital, Durban, in 2013.^14^ The omission of BI-RADS classifications, the absence of VAB sampling technique (only CNB was included) and the lack of specimen radiography for calcified abnormalities are viewed as study limitations. We conducted this study to evaluate the radiological-pathological concordance of stereotactic breast biopsies in a referral hospital breast imaging unit in South Africa with the objectives of describing the mammographic findings and histological diagnosis in patients who underwent stereotactic breast biopsy and evaluating the proportion of malignancy in each BI-RADS category.

### Research methods and design

Stereotactic breast biopsies performed at the Charlotte Maxeke Johannesburg Academic Hospital mammography unit in Johannesburg, South Africa, between 01 August 2016 and 31 July 2019 were retrospectively reviewed. Only lesions biopsied using stereotactic techniques were documented. For this article, the term ‘stereotactic biopsies’ includes both traditional stereotaxis and with tomosynthesis guidance. The vast majority of patients were seen and investigated by one of two experienced radiologists (with 17- and 25-years’ experience, respectively).

All potential biopsy patients underwent tomosynthesis mammographic imaging as well as bilateral breast and axilla ultrasound in order to plan the most appropriate biopsy method. Stereotactic biopsy methods included prone stereotactic (PS) biopsy (with the MultiCare Platinum Prone Breast Biopsy Table) and erect DBT-guided biopsy (with the Selenia Dimensions Mammography System). Sampling methods included CNB (usually using a 14-gauge needle) or VAB (using a 9-gauge vacuum probe). If the target lesion included calcifications, post-procedure specimen radiography was performed to confirm their presence prior to radiographic marker insertion (which was then confirmed with a post-procedure mammogram). During the time of the data collection, the fourth version of the BI-RADs lexicon was in use by the radiologists in the unit. As a result, the calcification morphology is described as benign, indeterminate or suspicious (and the definitions of each category are taken from that version of the lexicon).

Stereotactic samples that showed more than one diagnosis on histology were classified based on the highest risk lesion. Benign findings included fibrocystic change, papillomas, fibroadenomatoid change, inflammatory changes (fat necrosis and mastitis), stromal fibrosis, adenosis/sclerosing adenosis, scar/surgical site changes, vascular proliferation and pseudoangiomatous stromal hyperplasia. In this study, high-risk lesions included lobular carcinoma in situ (LCIS), radial scar/complex sclerosing lesion, intra-ductal papilloma, flat epithelial atypia (FEA), fibrocystic change with atypia, atypical ductal hyperplasia (ADH) and atypical lobular hyperplasia (ALH). Malignant lesions included ductal carcinoma in situ (DCIS), invasive carcinoma of no specific type (NST), other invasive carcinomas (where type is specified) and other malignancies. According to nuclear grade, DCIS is classified as low-, intermediate- or high-grade.^15^ Considering its malignant potential and proclivity for local recurrence (albeit over a longer period of time), low-grade DCIS has been included in the malignancy category.^15,16^

Patient data were collected from the biopsy procedural recording sheets, breast imaging records, the Picture Archiving and Communication System (PACS) and the National Health Laboratory Service (NHLS). Demographic information, biopsy indication, mammography findings (including BI-RADS category) and histological findings were recorded. For patients with more than one lesion, who underwent multiple stereotactic biopsies, each lesion was documented separately. Follow-up surgical excision histology was reviewed, when available. Data were transferred to a data-capturing device (Microsoft Excel computer software) and analysed. Further analysis was conducted using SAS^®^ version 9.2. Descriptive statistics, namely, frequencies and percentages, were calculated for categorical data. Malignancy rates per BI-RADS category were calculated using cross tabulations.

## Ethical considerations

An application for full ethical approval was made to the Human Research Ethics Committee (Medical) of the University of the Witwatersrand and ethics consent was obtained on 19 February 2020 (approval number: M191197). All procedures performed were in accordance with the ethical standards of the institutional research committee.

### Results

A total of 146 stereotactic breast biopsies were considered for inclusion. Fourteen biopsies were excluded because of incomplete records. One biopsy was identified as a repeat biopsy (with the same histological diagnosis). Therefore, a total of 131 biopsies from 123 patients were included in the study. The mean age was 59.1 years (standard deviation 12.0 and range 33–84 years). All samples were retrieved from female patients. All patients were selected for biopsy using the BI-RADS guidelines. The overwhelming majority were category 4 and 5 lesions.

Indications for biopsy and selected imaging features are presented in Table 1. A large majority (79.4%) of patients had findings detected on screening or surveillance studies (i.e. on asymptomatic breasts). Ultimately, most of the malignant lesions (72.5%) also came from patients with no breast-related symptoms. There was a marginal predominance of the DBT-guided biopsy technique, comprising 59.5% of samples. Only two CNBs were performed (both with stereotactic guidance); the rest (98.5%) were performed with VAB. Most lesions (62.6%) demonstrated some form of calcification. Five (3.8%) BI-RADS 3 lesions were biopsied. In three of these cases, the patients had an additional BI-RADS 4a lesion in the contralateral breast and underwent bilateral breast biopsies. The remaining two cases were performed at the patients’ request (one had a strong family history of breast cancer).

**TABLE 1:** Lesion characteristics as per breast imaging reporting and data system category of the stereotactic breast biopsies.

Lesion characteristics	BI-RADS										Total (*n* = 131)	
	3 (*n* = 5)		4a (*n* = 18)		4b (*n* = 69)		4c (*n* = 27)		5 (*n* = 12)			
	*n*	%	*n*	%	*n*	%	*n*	%	*n*	%	*n*	%
**Asymptomatic**	5	100.0	18	100.0	52	75.4	21	77.8	8	66.7	104	79.4
**Laterality**									
Right	3	60.0	9	50.0	31	44.9	16	59.3	7	58.3	66	50.4
Left	2	40.0	9	50.0	38	55.1	11	40.7	5	41.7	65	49.6
**Breast density**									
Type A	-	0.0	3	16.7	13	18.8	2	7.4	2	16.7	20	15.3
Type B	2	40.0	9	50.0	35	50.7	12	44.4	6	50.0	64	48.9
Type C	3	60.0	6	33.3	21	30.4	13	48.1	4	33.3	47	35.9
Type D	-	0.0	-	0.0	-	0.0	-	0.0	-	0.0	-	0.0
**Location**									
UOQ	4	80.0	8	44.4	34	49.3	8	29.6	3	25.0	57	43.5
UIQ	-	0.0	5	27.8	16	23.2	10	37.0	5	41.7	36	27.5
LIQ	-	0.0	3	16.7	8	11.6	4	14.8	2	16.7	17	13.0
Other†	1	20.0	2	11.1	11	15.9	5	18.5	2	16.7	21	16.0
**Calcification present**	3	60.0	13	72.2	40	58.0	17	63.0	9	75.0	82	62.6
**Calcification morphology**									
Benign	2	40.0	2	11.1	-	0.0	-	0.0	-	0.0	4	3.1
Indeterminate	1	20.0	10	55.6	24	34.8	5	18.5	2	16.7	42	32.1
Suspicious	-	0.0	1	5.6	16	23.2	12	44.4	7	58.3	36	27.5
**Calcification distribution**									
Regional	-	0.0	1	5.6	-	0.0	1	3.7	-	0.0	2	1.5
Cluster	-	0.0	10	55.6	30	43.5	9	33.3	6	50.0	55	42.0
Segmental	1	20.0	1	5.6	7	10.1	4	14.8	2	16.7	15	11.5
**Mass-like abnormality**	-	0.0	1	5.6	5	7.2	2	7.4	3	25.0	11	8.4
**Architectural distortion**	4	80.0	5	27.8	27	39.1	6	22.2	5	41.7	47	35.9
**Asymmetry**	1	20.0	2	11.1	6	8.7	3	11.1	-	0.0	12	9.2

Note: Percentages expressed are those of the corresponding column. Summation of column percentages will not equal 100 as each BI-RADS lesion displays multiple characteristics.

BI-RADS, Breast Imaging Reporting and Data System; UOQ, upper outer quadrant; UIQ, upper inner quadrant; LIQ, lower inner quadrant.

† , Includes lower outer quadrant, mid-lateral, mid-medial, mid-lower, mid-upper, axillary tail and retroareolar.

**FIGURE 1:**
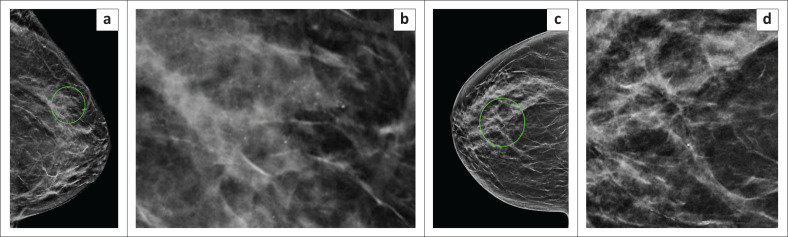
Examples of lesions with radiological-pathological discordance based on stereotactic biopsy histology, which were thereafter upgraded. (a) Craniocaudal view and (b) magnified image demonstrating grouped amorphous and pleomorphic microcalcifications in the outer left breast. These were new compared with previous imaging and categorised as breast imaging reporting and data system (BI-RADS) 4c. Stereotactic biopsy yielded benign histology, and this was considered discordant to imaging. Repeat stereotactic biopsy yielded low-grade ductal carcinoma in situ. (c) Right craniocaudal view demonstrating architectural distortion in the central retroareolar region of the right breast. (d) Magnified image showing the associated microcalcifications. This was categorised as BI-RADS 4c. Stereotactic biopsy histology showed benign fibrocystic changes only. Following a multidisciplinary team meeting, a wide-local excision was performed, and histology yielded fat necrosis.

**FIGURE 2:**
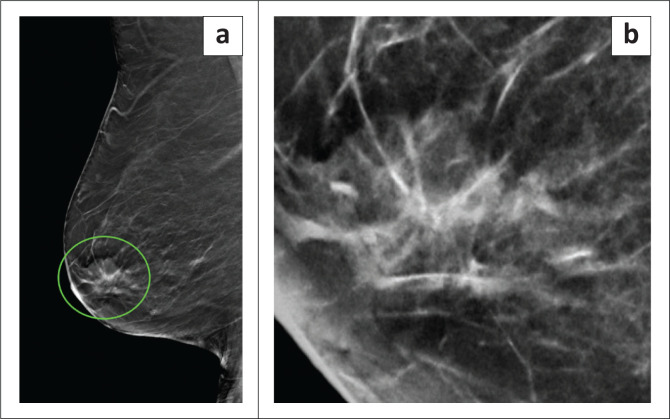
A lesion with radiological-pathological discordance based on stereotactic biopsy histology. (a) Mediolateral oblique reconstructed image from digital breast tomosynthesis revealing a spiculated mass in the retroareolar region of the right breast. (b) On close inspection, associated amorphous microcalcifications can be seen. This was categorised as breast imaging reporting and data system 5. Stereotactic histology revealed a fibroepithelial lesion. Wide-local excision after hook-wire placement yielded only benign breast tissue.

The distribution of benign, high-risk, and malignant histological diagnosis in each BI-RADS category is shown in Table 2, alongside the positive predictive value (PPV) for malignancy for each BI-RADS category (as well as the expected PPV as per the BI-RADS guidelines). For the purposes of comparison with the BI-RADS guidelines, the PPV includes only malignant lesions as positive histology (i.e. does not include high-risk lesions). Table 3 shows the range of histological findings in each BI-RADS category. In four lesions (3.1% of the total sample population), the radiologist deemed the imaging findings and histology results discordant (radiological-pathological discordance). Three of the four lesions were BI-RADS 4c with benign histology on stereotactic biopsy. The first case had a repeat biopsy showing low-grade DCIS (Figure 1a and b), the second case had a wide-local excision showing fat necrosis (Figure 1c and d) and the third case opted for a bilateral mastectomy for confirmed breast cancer in the contralateral breast (not shown, mammogram performed at another facility). The surgical histology revealed invasive carcinoma in both breasts. The remaining fourth lesion was categorised as BI-RADS 5, and the stereotactic biopsy revealed a fibroepithelial lesion (Figure 2). This was considered radiological-pathological discordant and wide-local excision was performed. The surgical specimen contained no neoplasm and histology showed benign breast tissue only. Of the 11 masses or mass-like abnormalities that were biopsied, more than half (*n* = 6, 54.5%) yielded high-risk or malignant histology. Of special note is that there was a solitary case of Kaposi sarcoma recorded amongst the malignant diagnoses.

**TABLE 2:** Distribution of histological diagnosis as per breast imaging reporting and data system category.

BI-RADS	Stereotactic biopsy histology			PPV		BI-RADS guidelines PPV %
	Benign	High-risk	Malignant	*n*	%	
**3 (*n* = 5)**						
***n***	5	0	0	0/5	0	> 0 but ≤ 2
**Row %**	100.0	-	-	-	-	-
**4a (*n* = 18)**						
***n***	13	3	2	2/18	11.1	> 2 to ≤ 10
**Row %**	72.2	16.7	11.1	-	-	-
**4b (*n* = 69)**						
***n***	46	2	21	21/69	30.4	> 10 to ≤ 50
**Row %**	66.7	2.9	30.4	-	-	-
**4c (*n* = 27)**						
***n***	16	1	10	10/27	37.0	> 50 to < 95
**Row %**	59.3	3.7	37.0	-	-	-
**5 (*n* = 12)**						
***n***	3	2	7	7/12	58.3	≥ 95
**Row %**	25.0	16.7	58.3	-	-	-
**Total (*n* = 131)**						
***n***	83	8	40	-	-	-
**Row %**	63.4	6.1	30.5	-	-	-

BI-RADS, Breast Imaging Reporting and Data System; PPV, positive predictive value.

**TABLE 3:** Histopathological findings in 131 stereotactic biopsies and frequency in each breast imaging reporting and data system category.

Histology finding	BI-RADS										Total	
	3		4a		4b		4c		5			
	*n*	%	*n*	%	*n*	%	*n*	%	*n*	%	*n*	%
**Benign (*n* = 83)**												
FCC	4	80.0	6	33.3	36	52.2	9	33.3	0	-	55	42.0
Papilloma	0	-	1	5.6	1	1.4	0	-	0	-	2	1.5
Fat necrosis	0	-	3	16.6	3	4.4	2	7.4	1	8.3	9	6.9
Mastitis	0	-	0	-	2	3.0	0	-	0	-	2	1.5
Benign fibroepithelial lesion	0	-	0	-	0	-	1	3.7	1	8.3	2	1.5
Benign breast tissue	1	20.0	3	16.6	4	5.8	4	14.8	1	8.3	13	9.9
**High-risk (*n* = 8)**												
ADH	0	-	2	11.1	1	1.4	0	-	1	8.3	4	3.0
Intra-ductal papilloma	0	-	0	-	0	-	1	3.7	1	8.3	2	1.5
Radial scar/CSL	0	-	1	5.6	0	-	0	-	0	-	1	0.8
Atypical apocrine metaplasia	0	-	0	-	1	1.4	0	-	0	-	1	0.8
**Malignant (*n* = 40)**												
DCIS	-	0.0	1	5.6	14	20.3	7	26.0	4	33.4	26	19.8
Invasive carcinoma NST	-	0.0	1	5.6	5	7.3	2	7.4	3	25.0	11	8.4
Invasive tubular carcinoma	-	0.0	0	-	1	1.4	0	-	0	-	1	0.8
Invasive lobular carcinoma	-	0.0	0	-	1	1.4	0	-	0	-	1	0.8
Kaposi sarcoma	-	0.0	0	-	0	-	1	3.7	0	-	1	0.8

**Total**	**5**	**100.0**	**18**	**100.0**	**69**	**100.0**	**27**	**100.0**	**12**	**100.0**	**131**	**100.0**

Note: Percentages expressed are those of the corresponding column.

BI-RADS, Breast Imaging Reporting and Data System; FCC, fibrocystic changes; ADH, atypical ductal hyperplasia; CSL, complex sclerosing lesion; DCIS, ductal carcinoma in situ; NST, no specific type.

For sensitivity and specificity, true-positive mammograms were defined as those with a BI-RADS 4 or 5 classification and a malignant or high-risk diagnosis on stereotactic biopsy histology. A true-negative mammogram was defined as a BI-RADS category 3 lesion with benign histology. False-positive mammograms were defined as any BI-RADS 4 or 5 lesions that yielded a benign histology result. False-negative mammograms were defined as a BI-RADS 3 lesion with a malignant or high-risk diagnosis on stereotactic biopsy histology. This study yielded an imaging assessment sensitivity of 100% (95% confidence interval [CI]: 93% – 100%) and a specificity of 6.0% (95% CI: 2% – 14%).

Of the 48 positive stereotactic biopsy results (including both high-risk and malignant lesions), 11 patients did not undergo surgery. They were either considered not suitable for surgery (for severe comorbid medical conditions or for the presence of metastatic disease), or the patient did not undergo surgery for personal reasons (defaulting follow-up or personally opting for conservative management). Of the remaining 37 lesions, follow-up data were not available for eight lesions. It is likely that these patients returned to their referring clinician at a different facility. Surgical histology was available for the remaining 29 lesions (29/37, 78.4%), which are presented in Table 4. Twenty lesions (67.9%) were concordant on stereotactic and surgical histology. Of the nine remaining lesions, 33.3% (3/9) were clear upgrades from DCIS to invasive carcinoma. The rest (66.7%, 6/9) were essentially ‘downgraded’, showing a greater degree of benignancy on surgical excision (see Table 4). When surgical excision histology reveals a histological diagnosis with greater degree of malignancy, the stereotactic biopsy result is considered an underestimation of the true lesion. Overall, the underestimation rate of histology obtained from stereotactic biopsies versus surgical excision was 10.3% (3/29).

**TABLE 4:** Comparison of stereotactic biopsy histology with surgical excision histology.

Histology results	Surgical excision histology							Total	
	Benign	Atypia†	Intra-ductal papilloma	ADH	LG/IG DCIS	HG DCIS	Invasive carcinoma	*n*	%
**Stereotactic biopsy histology**									
Benign	0	0	0	0	0	0	0	0	-
Atypia†	1	0	0	0	0	0	0	1	3.5
Intra-ductal papilloma	0	0	1	0	0	0	0	1	3.5
ADH	2	0	0	0	0	0	0	2	6.9
LG/IG DCIS	1	0	0	0	5	0	1	7	24.1
HG DCIS	0	0	0	0	1	6	2	9	31.0
Invasive carcinoma	1	0	0	0	0	0	8	9	31.0

**Total**									
***n***	**5**	**0**	**1**	**0**	**6**	**6**	**11**	**29**	-
**%**	**17.2**	**-**	**3.5**	**-**	**20.7**	**20.7**	**37.9**	**-**	**100.0**

Note: Percentages expressed are of the total 29 lesions.

ADH, atypical ductal hyperplasia; LG, low-grade; IG, intermediate grade; HG, high-grade; DCIS, ductal carcinoma in situ.

† , atypical apocrine metaplasia.

This study included 10 patients with high-risk lesions on stereotactic biopsy. Two of the 10 patients had concurrent malignant findings on the same stereotactic biopsy sample and were managed as malignancies. Of the remaining eight patients with high-risk lesions, follow-up data were available for six patients (75.0%). Two of these six patients were managed conservatively (one had ADH only and the other had an intra-ductal papilloma). Both were reported as stable on follow-up imaging at 6 months and thereafter returned to annual follow-up. Both cases showed radiological stability up to the date of writing. The remaining four patients with high-risk lesions underwent surgical excision, yielding concordance in one case and discordance in the other three cases. The three discordances included one case of atypical apocrine metaplasia and two cases of ADH on stereotactic biopsy, all yielding fibrocystic change on surgical excision (Table 4). Following benign surgical results, all discordant cases were planned for 6-month imaging follow-up, and thereafter to return to annual follow-up. Follow-up data were available for two of the three discordant cases. One case presented for her 6-month visit and the other only presented at 1 year; however, both showed imaging stability, with no new suspicious findings two years after biopsy. At the time of writing, the third case had not returned to our unit for follow-up.

### Discussion

Stereotactic breast biopsies are performed on lesions with subtle imaging findings, no palpable mass and no sonographic correlate.^7,8,9^ They are performed in the hope of early breast cancer detection and better patient outcomes.^7,8,9^ The role of radiologists in breast imaging is to correctly identify high-risk lesions and therefore select patients who require further investigation.

In this study, many stereotactic breast biopsies that resulted in a malignant diagnosis occurred in asymptomatic women. This highlights the value of screening and surveillance programmes for breast cancer. Currently, in South Africa, an organised national screening programme does not exist. However, there are centres where opportunistic screening is performed.^17^ International guidelines continue to advocate for the role of screening and surveillance in the reduction of breast cancer deaths.^18,19^

When comparing the imaging findings with early studies, we found several similarities. Most of the target lesions had calcifications and were classified as BI-RADS category 4.^20,21,22,23,24,25^ A later study making use of the BI-RADS 4 subcategories reported 4a as their dominant subcategory, in comparison to our study in which 4b was the most prevalent.^23^

**FIGURE 3:**
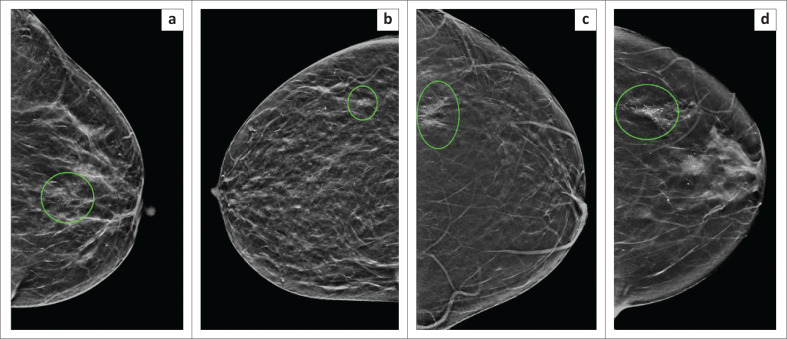
Examples of typical lesions that were suspicious for malignancy on breast imaging and confirmed malignancy on stereotactic biopsy histology. Craniocaudal views demonstrating (a) grouped pleomorphic microcalcifications in the central retroareolar region of the left breast, which showed a high-grade ductal carcinoma in situ on histology, (b) a small group of pleomorphic microcalcifications in the outer right breast, which revealed high-grade ductal carcinoma in situ on histology, (c) architectural distortion with associated grouped pleomorphic microcalcifications in the outer left breast. Histology yielded an invasive carcinoma of no specific type, (d) Two groups of suspicious microcalcifications in the left breast. The central retroareolar group of microcalcifications had a positive ultrasound correlate and was sampled by ultrasound-guided biopsy. The group of pleomorphic microcalcifications in the outer breast (circled) yielded invasive carcinoma of no specific type on stereotactic biopsy histology.

Calcifications are well-recognised targets for stereotactic biopsies.^24^ Many cases of typical suspicious calcifications resulted in positive diagnoses of malignancy (Figure 3). Indeterminate calcifications, although mainly occurring in category 4, featured across all BI-RADS categories included in the study (see Table 1). When categorised as BI-RADS 3 (in a solitary case), there were no associated imaging findings and the lesion yielded benign histology. One of the two BI-RADS 5 lesions with indeterminate calcifications also had other concerning imaging findings for malignancy (an associated spiculate mass and architectural distortion) and histology revealed an intra-ductal papilloma. In the other case of a BI-RADS 5 lesion, the indeterminate calcifications were reported in a clustered distribution and yielded DCIS on stereotactic histology.

This widespread distribution of essentially ‘uncertain’ calcifications emphasises the diagnostic challenge that calcifications represent. Digital breast tomosynthesis is superior in detecting non-calcified mammographic abnormalities for biopsy. The higher proportion of architectural distortions in this study may be explained by our routine use of DBT, in contrast to Cupido et al.’s study, where DBT was not employed and no architectural distortions were reported.^14^ This is further supported by Rochat et al., who reported architectural distortion in 2.0% of their digital mammography stereotactic biopsies versus 17.7% in their DBT-guided biopsies.^25^

Mass-like lesions are also targeted with stereotaxis if there is no sonographic correlate. Fifty-five percent (6/11) of the mass-like abnormalities biopsied in this study resulted in positive histology (malignancies or high-risk lesions). In comparison to Mendez et al., with 25.0%, and Bohan et al., with 12.5%, our malignancy rate amongst mass-like lesions is high.^21,26^ However, there were no circumscribed masses in our study (they were all described as having a spiculated or indistinct margin). When excluding the circumscribed masses from the study by Mendez et al., the malignancy rate amongst mass-like lesions is 66.0%.^21^ Nonetheless, the high malignancy rate in our study warrants a high degree of suspicion when confronting mammographically detected mass-like lesions in our setting.

Looking at the mammograms of patients that underwent stereotactic core biopsies in our unit (*n* = 131), the BI-RADS suspicion for malignancy (category 4 or 5) was 100% sensitive, but with a very low specificity of 6.0%. In other words, every lesion that resulted in a histological diagnosis of a high-risk lesion or a malignancy had a mammogram that was assessed as either suspicious (category 4) or highly suspicious (category 5) for malignancy. The sensitivity is expected to be high in a screening tool like mammography, and this finding is reassuring when using BI-RADS in our setting. In a comparable study that looked at 947 stereotactic VABs, Mendez et al. reported a sensitivity of 94.7% and a specificity of 18.8%.^21^

Our particularly low specificity is influenced by the high number of false positives (lesions assessed as BI-RADS 4 or 5 that yielded benign histology). The patients that were likely to be included in the study were patients who had indeterminate lesions, like architectural distortions and indeterminate calcifications. Bahl et al. demonstrated a PPV of 74.5% for malignancy in 369 cases of architectural distortions with sonographic correlates. However, when architectural distortion occurred without a positive sonographic correlate (like in our study), Bahl et al. showed that the likelihood of it representing malignancy was only 27.9%.^27^ The other contributing factor was the low number of true negatives (mammographically benign lesions that resulted in benign histology) – in other words, BI-RADS 3 lesions that were proven benign on histology. We had less than 4.0% BI-RADS 3 lesions in our study (5/131). Breast Imaging Reporting and Data System 3 lesions are not usually managed with biopsy unless specifically requested by the patient and/or clinician.^5,21^

**FIGURE 4:**
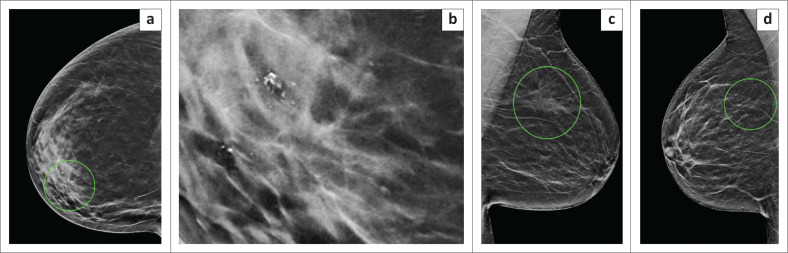
Examples of lesions that were suspicious for malignancy on breast imaging, but yielded benign histology. (a) Craniocaudal view and (b) magnified image showing architectural distortion and suspicious microcalcifications in the inner right breast. Histology revealed fat necrosis, (c) Mediolateral oblique view indicating a poorly circumscribed mass with spiculated margins in the upper left breast, associated with architectural distortion and amorphous microcalcifications. Histology revealed an intra-ductal papilloma, (d) Mediolateral oblique view demonstrating architectural distortion in the upper right breast which yielded benign breast tissue on histology.

The BI-RADS guidelines were developed to universalise and standardise reporting around the world. As part of the guidelines, an expected range of malignancy is given for each category.^5^ In our results, although there is a stepwise increase in the proportion of malignancy from category 3 to 5, the proportions in category 4c and especially in category 5, were lower than the range in the BI-RADS guidelines (see Table 2). A big contributor was our BI-RADS 5 lesions with benign histology. On closer inspection of these false-positive cases, the imaging features were suspicious and included: a fibroadenoma with architectural distortion and suspicious calcifications (Figure 2), fat necrosis with architectural distortion and suspicious calcifications (Figure 4a and 4b) and an intra-ductal papilloma with an ill-defined density, architectural distortion and indeterminate calcifications (Figure 4c). A single BI-RADS 5 lesion yielded completely benign breast tissue (Figure 4d). These cases contributed to the low specificity of our stereotactic biopsies.

High-risk lesions are a controversial topic amongst pathologists, breast surgeons and radiologists alike.^28^ This lack of consistency is apparent in the literature. While there is consensus on the malignant potential of ADH, there remains uncertainty about the clinical importance and management of intra-ductal papillomas, radial scars, ALH and LCIS.^28,29^ Underestimation rates are used to report discordances between surgical excision and stereotactic biopsy histology if the surgical histology shows a greater degree of malignancy than the stereotactic biopsy. Along with DCIS, high-risk lesions as mentioned above are listed as contributors to underestimation rates.^20,23,26,30,31^ Our DCIS underestimation rate of 10.3% (*n* = 3) is comparable to results from other studies (Table 5). All three of our cases involved the upgrading of DCIS to invasive carcinoma. Pieters et al. conducted a study assessing underestimation rates in South Africa^32^ and reported a DCIS underestimation rate of 12.5%. However, as with our study, they also had a small number of lesions in this group and therefore this should be interpreted with caution. There were five lesions in our study that showed more aggressive histology on stereotactic biopsy than the surgical excision. One possible explanation is that these ‘downgraded’ lesions were completely removed by the stereotactic VAB. The other consideration is that different areas were sampled during surgery.

**TABLE 5:** Comparison table of studies evaluating radiological-pathological discordance or histological underestimation in stereotactic breast biopsies.

Study	Year	Site	No. of biopsies	Method	Total malignancy (%)	Radiological-pathological discordance			Underestimation rates
						Discordances (%)	*n*	Sample population	
Pfarl et al.	2002	Austria	318	SVAB	63.2	4.10	13	318	12.1% (DCIS)
Liberman et al.	2002	United States	800	SVAB	26.8	2.30	18	800	14.2% (DCIS)
Ciatto et al.	2007	Australia	1388	SVAB	N/R	4.40†			23.4% (overall)
Jackman et al.	2009	United States	1280	SVAB	38.2	1.30	16	1280	13% (high-risk lesions)
Venkataraman et al.	2012	United States	912	SVAB	22.4	0.85	4	471‡	12.2% (DCIS)
Cupido et al.	2013	South Africa	67	SCNB	20.9	10.40	7	67	N/R
Heller et al.	2016	United States	1861	SVAB	29.1	1.20	23	1861	23.3% (overall of discordant lesions)
Pieters et al.	2016	South Africa	158	SVAB	28.5		N/R		N/R
Rochat et al.	2019	United States	1405	SVAB/DBT-VAB	26.3	3.10	43	1405	6.3% (overall)
This study	2022	South Africa	131	SCNB/SVAB/DBT-VAB	30.5	3.10	4	131	10.3% (DCIS)

SCNB, stereotactic core needle biopsy; SVAB, stereotactic vacuum-assisted biopsy; DBT-VAB, digital breast tomosynthesis-guided vacuum-assisted biopsy; N/R, not reported.

† , numerical/denominator not available.

‡ , Benign core biopsies.

Several studies addressing radiological-pathological concordance or underestimation rates in stereotactic breast biopsies are shown in Table 5.^14,25,32,33,34,35,36,37,38^ Accurately comparing studies is difficult. Studies dedicated specifically to investigating the radiological-pathological concordance do not always utilise the BI-RADS categories and there is a wide range of sample sizes and biopsy techniques. Radiological-pathological concordance has also been assessed by correlating malignancy rates with specific imaging features, rather than BI-RADS categories.^14,24^ The categorisation of high-risk lesions is notoriously inconsistent.^28^ In our study, we had a small sample size, but the overall malignancy rate was comparable to most other studies (Table 5). Our radiological-pathological discordance rate of 3.1% is comparable to the findings of international studies. The DCIS underestimation rate for our study is comparable to that of local and international studies, where specific DCIS underestimation rates are reported or may be calculated, ranging from 10.3% to 14.2%.^33,34,37^

## Study limitations

All biopsies were performed at a single tertiary breast imaging centre. Most of the mammograms and biopsies were interpreted and performed by one of the two experienced radiologists. This is not representative of all clinical service centres.

In our study, histology from stereotactic biopsy samples was used as the gold standard for sensitivity and specificity as not all patients had surgical excision or long-term follow-up.

### Conclusion

The stereotactic biopsies detected cancer in predominantly asymptomatic women during screening and surveillance studies. This study showed that stereotactic breast biopsies performed in a referral hospital in South Africa were very sensitive, but not very specific. We produced a radiological-pathological concordance that is comparable to international studies, with similar discordance and underestimation rates.
